# Adaptive islet biology and comprehensive incretin physiology: Emerging perspectives from Asia and Europe

**DOI:** 10.1111/jdi.70378

**Published:** 2026-06-25

**Authors:** Takaaki Murakami, Daniela Nasteska, David J. Hodson, Yuji Yamazaki, Daisuke Yabe, Yutaka Seino

**Affiliations:** ^1^ Department of Diabetes, Endocrinology and Nutrition Kyoto University Graduate School of Medicine Kyoto Japan; ^2^ Oxford Centre for Diabetes, Endocrinology and Metabolism (OCDEM), NIHR Oxford Biomedical Research Centre, Bukhman Centre for Research Excellence in Type 1 Diabetes, Churchill Hospital, Radcliffe Department of Medicine University of Oxford Oxford UK; ^3^ Kansai Electric Power Hospital Osaka Japan; ^4^ Kansai Electric Power Medical Research Institute Osaka Japan

## Abstract

Adaptive islet biology and incretin physiology provide a framework linking diverse diabetes phenotypes, with advances in β‐cell stress adaptation, intra‐islet communication, receptor‐resolved biology, and β‐cell mass imaging. By integrating clinical perspectives from Asia and Europe with multiscale islet‐cell biology, these insights may support mechanism‐based and more targeted therapeutic approaches.

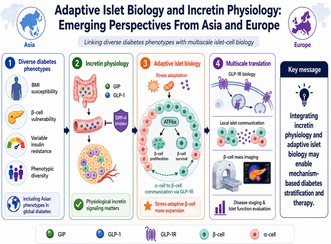

Type 2 diabetes phenotypes vary substantially within and across regions. In contrast to the specifics of type 2 diabetes in Caucasians, East Asian populations demonstrate a clinical pattern in which susceptibility to type 2 diabetes can occur at relatively lower body mass index, with impaired insulin secretion playing a prominent role. In an increasingly globalized world, Europe, North America, and other multiethnic societies are home to a significant number of individuals of Asian ancestry and many other populations with diverse combinations of insulin secretory capacity, insulin resistance, and incretin responsiveness. Therefore, understanding the heterogeneity of diabetes phenotypes across different ancestral backgrounds, including Asian populations, is essential for developing treatment strategies tailored to distinct pathophysiological profiles.^1^

Advances in islet‐cell and incretin biology now allow this clinical concept to be revisited at much higher biological resolution (Figure 1). The question is no longer simply whether β‐cells can secrete enough insulin. Rather, it is how β‐cells and other islet endocrine cells sense metabolic stress, adapt their mass and function, communicate with neighboring cells, and eventually fail. In parallel, incretin biology has moved beyond the measurement of gut hormone secretion and glucose‐lowering drug effects toward receptor localization, ligand trafficking, intra‐islet communication, and molecular imaging. Recent discussions among investigators from Asia and Europe, including active participation by young researchers at the Young Researcher Initiated Focus Group Meeting on Islet‐Cell and Incretin Biology 2026 in Osaka, further highlighted the need to anchor diverse diabetes phenotypes in contemporary advances in islet‐cell and incretin biology.

**Figure 1:**
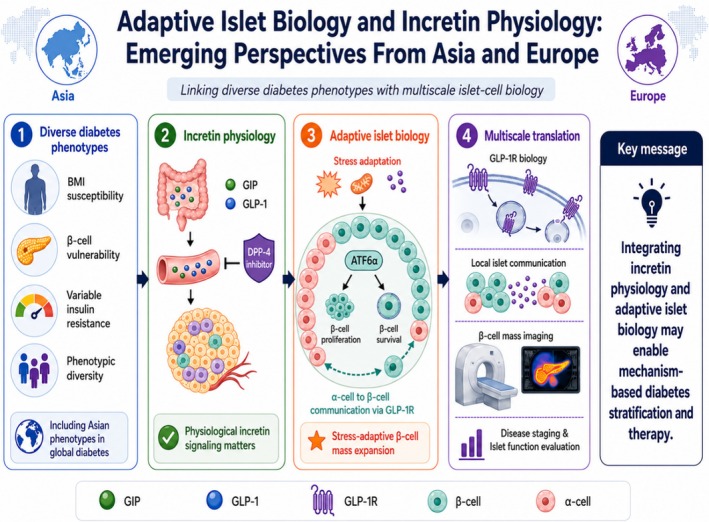
Adaptive islet biology and incretin physiology provide a framework linking diverse diabetes phenotypes, including Asian diabetes phenotypes, with advances in β‐cell stress adaptation, intra‐islet communication, receptor‐resolved biology, and β‐cell mass imaging. By integrating clinical perspectives from Asia and Europe with multiscale islet‐cell biology, these insights may support mechanism‐based diabetes stratification and inform more targeted therapeutic approaches. ATF6α, activating transcription factor 6 alpha; BMI, body mass index; DPP‐4, dipeptidyl peptidase‐4; GIP, glucose‐dependent insulinotropic polypeptide; GLP‐1, glucagon‐like peptide‐1; GLP‐1R, glucagon‐like peptide‐1 receptor.

A major advance is the renewed appreciation of endogenous incretin physiology. Dipeptidyl peptidase‐4 (DPP‐4) inhibitors have been especially important in diabetes care, in part because they enhance endogenous incretin action in patients whose diabetes is linked to impaired insulin secretion. Kubota‐Okamoto *et al*.^2^ recently showed that endogenous glucose‐dependent insulinotropic polypeptide (GIP) signaling is indispensable for dipeptidyl peptidase‐4 inhibitor‐mediated metabolic control in mice. This finding is conceptually important because it emphasizes that DPP‐4 inhibition cannot be understood solely as a glucagon‐like peptide‐1 (GLP‐1) enhancing strategy. Instead, its therapeutic effect reflects an integrated incretin network in which endogenous GIP signaling has an essential role, at least in the experimental setting studied.

This insight is timely in the current era of GLP‐1 receptor agonists, dual GLP‐1/GIP receptor agonists, and emerging multi‐agonist therapies. Pharmacological incretin biology is rapidly expanding, but the physiological roles of endogenous incretins remain fundamental. Understanding the timing and tissue width of endogenous incretin signals, how they are altered in diabetes, and how they interact with β‐cell stress programs will be essential for translating incretin‐based therapies into more mechanism‐based treatment strategies.

Another major advance is the recognition that β‐cell mass expansion is not a uniform process. Otani *et al*.^3^ demonstrated that activating transcription factor 6α, a key branch of the unfolded protein response, governs stress‐adaptive pancreatic β‐cell mass expansion by coordinating β‐cell proliferation and survival. This work reframes endoplasmic reticulum stress signaling not only as a pathway of cellular injury but also as a mechanism that supports adaptive β‐cell expansion under metabolic demand. The implication is that β‐cell stress responses should not be interpreted simply as markers of impending failure but rather as essential homeostatic mechanisms that maintain function. Under appropriate biological contexts, stress‐response pathways may contribute to the maintenance or expansion of β‐cell mass.

This distinction has important implications for diverse diabetes phenotypes. It introduces the concept of plasticity in β‐cell stress adaptation, which either sustains normal islet function or stands in the background of the direct clinical manifestations—reduced insulin secretory capacity, postprandial hyperglycemia, and responsiveness to incretin‐based therapies. Adopting this concept into research and clinical practice suggests that future therapies should aim not only to stimulate insulin secretion but also to preserve β‐cell identity, survival, and regenerative capacity.

Incretin biology itself has entered a receptor‐resolved era. Recent advances in chemical biology, imaging, and cellular physiology have enabled incretin receptors to be studied with increasing spatial and temporal precision. A particularly important conceptual development is that incretin receptors may participate in local islet‐cell communication. Tong *et al*.^4^ showed that localized GLP‐1 receptor pre‐internalization directs pancreatic α‐cell to β‐cell communication. This finding challenges the traditional view that incretin action is primarily an endocrine process in which gut‐derived hormones stimulate β‐cell insulin secretion. Instead, GLP‐1 receptor localization and trafficking may shape spatially restricted communication within the islet microenvironment.

This broader view also changes how α‐cells should be considered in diabetes biology. α‐cells are no longer viewed merely as glucagon‐secreting cells that oppose insulin action. They are increasingly recognized as active participants in islet adaptation, metabolic stress responses, and β‐cell regulation. Changes in α‐cell survival, function, and communication with β‐cells may contribute to diabetes pathophysiology in ways that cannot be captured by glucagon levels alone. Thus, diabetes should be considered a disorder of coordinated islet‐cell adaptation rather than isolated β‐cell failure.

A further advance is the development of non‐invasive β‐cell mass imaging as a bridge between molecular biology and clinical staging. Sakaki *et al*.^5^ reported that quantitative β‐cell mass imaging could refine disease staging and glycemic control in type 1 diabetes. By using GLP‐1 receptor‐targeted imaging to estimate β‐cell mass, this work introduces a cell‐based imaging measure into a field that has long relied on glucose levels, C‐peptide, and autoantibodies as indirect indicators of disease state. Although C‐peptide remains an indispensable functional marker, it reflects β‐cell function under specific physiological conditions, whereas β‐cell mass imaging aims to assess β‐cell quantity more directly.

The ability to distinguish β‐cell mass from β‐cell function may allow more precise disease staging, better identification of therapeutic windows, and improved evaluation of interventions designed to preserve or restore β‐cells. More broadly, GLP‐1 receptor‐targeted imaging links cellular incretin receptor biology with patient‐level diabetes phenotyping. The same receptor biology that can be studied at the level of ligand binding, receptor localization, and intra‐islet communication can also be exploited to visualize β‐cell‐related tissue in humans. This multiscale connection is likely to become increasingly important in type 1 diabetes, insulin secretion‐impaired type 2 diabetes, and other conditions in which β‐cell reserve determines clinical trajectory.

Taken together, these advances suggest a new framework for islet‐cell and incretin biology. Diverse diabetes phenotypes, including Asian diabetes phenotypes, underscore the clinical importance of β‐cell vulnerability and impaired insulin secretion. Modern β‐cell biology reveals that β‐cell failure reflects the disruption of stress‐adaptive programs that control proliferation, survival, identity, and function. Incretin biology, once centered on gut hormone secretion and glucose lowering, and GLP‐1‐skewed, now encompasses endogenous GIP signaling, receptor localization, ligand trafficking, intra‐islet communication, and drug target mapping. α‐cell biology adds another layer, emphasizing that islet adaptation involves multiple endocrine cell types and that β‐cell (dys)function cannot be fully understood in isolation. Finally, β‐cell mass imaging provides a route back to the clinic, allowing cellular processes to be connected with disease staging in living humans.

The field is therefore moving from a secretion‐centered model to an adaptation‐centered model of diabetes. In this model, diabetes develops when islet cells can no longer adequately adapt to metabolic, immune, circadian, and mitochondrial disturbances, or to endoplasmic reticulum stress. Incretin‐based therapies may be most powerful when understood not only as insulin secretagogues, but also as modulators of islet‐cell communication, metabolic resilience, and β‐cell survival. This perspective is particularly relevant to diabetes phenotypes characterized by limited β‐cell reserve, but it has broad implications across regions and populations.

Future progress will require integration across disciplines, regions, and populations. Our modern, multinational societies require an in‐depth understanding of the diversity of diabetes pathophysiology, including Asian diabetes phenotypes, and the development of optimal therapeutic strategies for each pathophysiological profile. Collaborative work across Asia and Europe is now providing tools for receptor visualization, islet‐cell physiology, stem‐cell models, molecular imaging, and human phenotyping. Sustained exchange and collaboration among young investigators in Asia and Europe will be particularly important for sharing concepts, technologies, and clinical perspectives across regions. Continuing such young investigator‐driven meetings will therefore be essential for fostering the next generation of collaborative islet‐cell and incretin biology. Building on this convergence, the next phase of diabetes investigation should aim to define, visualize, and therapeutically support adaptive islet‐cell biology.

In conclusion, recent advances in islet‐cell and incretin biology provide a framework for understanding diabetes as a disorder involving cellular adaptation, communication, and altered cell mass. This perspective connects the clinical diversity of diabetes, including non‐obese type 2 diabetes characterized by impaired insulin secretion in Asian populations, with contemporary molecular and imaging‐based approaches. By integrating endogenous incretin physiology, β‐cell stress adaptation, α‐cell–β‐cell communication, receptor‐resolved pharmacology, and β‐cell mass imaging, the field is moving toward more mechanism‐based staging, prevention, and treatment of diabetes on a global scale.

## Disclosure

D.J.H. has filed patents related to incretin receptor chemical probes (WO2024133236A3) and diabetes therapy (WO2024062254A1 and WO2025191276A1). D.J.H. receives licensing revenue from Celtarys Research for the provision of incretin receptor chemical probes. D.J.H. receives research funding from Amgen Inc. D.Y. received clinically commissioned/joint research grants from Novo Nordisk, Ono Pharmaceutical, Taisho Pharmaceutical, Terumo, and Arklay. D.Y. also received consulting or speaker fees from Sumitomo Dainippon Pharma, Boehringer Ingelheim, Astellas Pharma, MSD, Novo Nordisk, Ono Pharmaceutical, Eli Lilly, and Takeda Pharmaceutical. The other authors declare no conflict of interest.

## Data Availability

Data sharing not applicable to this article as no datasets were generated or analysed during the current study.
